# From G tolerance to physiological state monitoring: mechanisms and adaptive protection

**DOI:** 10.3389/fphys.2026.1883478

**Published:** 2026-07-22

**Authors:** Xiaowei Wang, Haowen Wu, Xiaoyi Yang, Zhenghui Wu, Yanzeng Zhao, Jiayi Bao, Ziyu Liu

**Affiliations:** 1 School of Energy and Power Engineering, Beihang University, Beijing, China; 2 Shanghai Aircraft Airworthiness Certification Center of CAAC, Shanghai, China; 3 School of Aeronautic Science and Engineering, Beihang University, Beijing, China

**Keywords:** anti-G straining maneuver, dynamic state assessment, G-induced loss of consciousness, high-G physiology, high-performance aviation, multimodal data fusion, physiological monitoring

## Abstract

G-induced loss of consciousness (G-LOC) remains a challenge in high-performance aviation because it can rapidly impair pilot functional capacity and compromise aircraft control. Traditional approaches have largely treated G-LOC as a physiological tolerance or threshold problem, emphasizing maximum +Gz tolerance, centrifuge-based assessment, and *post hoc* identification of loss of consciousness. However, operational G-LOC risk is more dynamic and depends on the interaction among +Gz magnitude, rate of onset, exposure duration, individual physiological reserve, and anti-G straining maneuver effectiveness. This review reframes G-LOC as a time-dependent physiological decompensation process rather than a discrete terminal endpoint. We synthesize evidence on the progression from compensated cardiovascular regulation to potential loss of consciousness, with particular attention to early functional degradation, including visual, cognitive, behavioral, and performance-related changes. We further discuss how mechanism-informed physiological monitoring may support adaptive protection strategies. This framework shifts G-LOC research from static tolerance assessment toward dynamic physiological state monitoring and individualized protection in high-performance aviation.

## 1 Introduction

G-induced loss of consciousness (G-LOC) remains a critical safety concern in high-performance aviation. During sustained or rapidly increasing +Gz acceleration, blood is displaced toward the lower extremities, reducing venous return, cardiac output, cerebral perfusion pressure, and ultimately cerebral oxygen delivery. If compensatory mechanisms fail, neural function may deteriorate and loss of consciousness may occur, often with limited warning and severe operational consequences, including transient pilot incapacitation and loss of aircraft control (Burton, 1988; Whinnery and Whinnery, 1990; Jones, 1991). Survey-based studies among fighter pilots and aircrew further indicate that G-LOC and related symptoms remain recurrent operational problems rather than isolated laboratory phenomena (**Table 1**).

**Table 1:** Prevalence of G-LOC and related symptoms reported in fighter pilot surveys.

Country/Air Force	Aircraft type/pilot group	Sample size	Non-LOC physiological symptoms	Reported G-LOC prevalence	Rapid-onset/fast-onset G-LOC	Reference
Australia (RAAF)	Operational fighter pilots	65	98% visual disturbances or cognitive impairment	9% reported at least one G-LOC event	Rapid onset possible during high-G maneuvers	Rickards and Newman, 2005
United Kingdom (RAF)	Fighter aircrew	809	32.2% A-LOC prevalence	14.8% reported G-LOC history	Rapid-onset cases reported during maneuvering flight	Slungaard et al., 2019
China (PLAAF)	Fighter pilots and aircrew	594	51% reported dizziness, visual disturbance, or other discomfort	8.2% reported G-LOC	Rapid G-LOC reported in high-G exposure	Cao et al., 2012
Turkey (Turkish Air Force)	Operational jet pilots	82	NR	7–10% reported previous G-LOC	Rapid onset during high-G maneuvers reported	Yilmaz et al., 1999
Multinational military aircrew	Mixed fighter aircraft	2259	NR	5–10% depending on subgroup	Rapid G-LOC described in operational flight	Green and Ford, 2006

Conventional G-LOC research has primarily focused on physiological tolerance, especially maximum tolerable +Gz levels derived from centrifuge testing. Although this approach has provided standardized information for pilot selection, training, and evaluation (Norsk, 1992; Whinnery and Whinnery, 1990), its value for risk prediction is limited. Real flight exposure is shaped not only by +Gz magnitude, but also by rate of onset, exposure duration, maneuver sequence, recovery interval, pilot actions, and individual physiological reserve (Benni et al., 2003a; Ryoo et al., 2004; Metzler, 2020; Blue et al., 2023). Therefore, fixed tolerance thresholds may fail to capture the time-dependent and individualized nature of G-LOC risk.

A further limitation is that operational impairment can develop before complete loss of consciousness. Visual disturbances, attentional decline, delayed response, impaired decision-making, and reduced situation awareness may occur while the pilot remains nominally conscious (Whinnery and Whinnery, 1990; Phillips and Hørning, 2015). These early-stage impairments are important because they can degrade flight-control performance before a clear incapacitation event is detected. Accordingly, G-LOC should be considered not only as a terminal physiological endpoint, but also as a progressive degradation process involving physiological regulation, neural function, and operational performance.

Recent advances in physiological monitoring provide new opportunities for detecting this degradation process. Cardiovascular signals, electroencephalography (EEG), near-infrared spectroscopy (NIRS), respiratory variables, electromyography (EMG), and behavioral indicators have all been explored as potential markers of G-induced physiological strain and functional impairment (Goodman et al., 2006; Sundblad et al., 2014). However, existing studies have often examined G-LOC mechanisms, physiological monitoring signals, predictive algorithms, and countermeasures as relatively separate topics. A clear integrative gap remains in linking the pathophysiological progression of G-LOC with multimodal monitoring indicators, individualized state assessment, and mechanism-informed protective strategies within a coherent physiological framework.

This review addresses this gap by synthesizing current evidence on G-LOC as a dynamic physiological state-transition process. Specifically, it aims to: (1) summarize the cardiovascular, cerebral, neural, visual, and cognitive mechanisms underlying G-LOC; (2) conceptualize G-LOC as a transition from compensated regulation to physiological decompensation and functional impairment; (3) evaluate cardiovascular, peripheral perfusion, cerebral oxygenation, autonomic, respiratory, electromyographic, and behavioral indicators including additional markers such as blood pressure variability (BPV) and electrodermal activity (EDA), as candidate leading indicators of G-LOC progression; and (4) discuss how multimodal data fusion, individualized modeling, AGSM feedback, adaptive training, anti-G protection, and pressure-breathing support may contribute to mechanism-informed physiological protection in high-performance aviation.

This review does not propose a new physiological mechanism of G-LOC, but integrates existing knowledge into a unified dynamic framework. Previous models have clarified the importance of +Gz magnitude, exposure duration, cerebral hypoperfusion, visual symptoms, and loss-of-consciousness thresholds. In contrast, the present framework emphasizes the trajectory by which a pilot may move from compensated cardiovascular regulation to cerebral vulnerability, functional degradation, A-LOC, G-LOC, and delayed recovery. It therefore shifts the analytical focus from endpoint tolerance assessment toward time-dependent physiological stability, cross-system interaction, candidate leading indicators, and intervention timing.

Accordingly, the framework should be interpreted as an integrative synthesis and operationally oriented conceptual framework, rather than as a new experimentally validated theoretical model. Its value lies in organizing existing evidence into a structure that can guide future research on physiological monitoring, individualized risk prediction, AGSM feedback, adaptive anti-G protection, and human–machine intervention. Further centrifuge and in-flight validation will be required before the framework can be used as an operational decision model.

## 2 Methods

### 2.1 Review design

This article was designed as a mechanism-oriented narrative review rather than a systematic review or meta-analysis. The purpose was not to generate pooled effect estimates or to experimentally validate a new physiological model, but to synthesize existing evidence on G-induced loss of consciousness (G-LOC) and organize it within a dynamic physiological-state framework. The review therefore integrates evidence from aerospace medicine, cardiovascular physiology, cerebral perfusion and oxygenation research, neurophysiology, physiological monitoring, multimodal data fusion, and anti-G protection technologies. The methodological approach was guided by general principles for narrative reviews, with emphasis on relevance, physiological plausibility, methodological transparency, and consistency with the known pathophysiology of +Gz exposure.

### 2.2 Information sources and search strategy

Relevant literature was identified through searches of PubMed/MEDLINE, Web of Science, Scopus, Google Scholar, IEEE Xplore, and aviation- or aerospace-medicine-related sources. Additional studies were identified by screening the reference lists of key articles and reviews. The search combined terms related to high-G exposure and loss of consciousness with terms related to physiological mechanisms, monitoring signals, prediction, and protection. Representative search terms included: “G-induced loss of consciousness, ” “G-LOC, ” “almost loss of consciousness, ” “A-LOC, ” “+Gz acceleration, ” “G tolerance, ” “high-G physiology, ” “cerebral perfusion, ” “cerebral oxygenation, ” “near-infrared spectroscopy, ” “NIRS, ” “fNIRS, ” “electroencephalography, ” “EEG, ” “heart rate variability, ” “blood pressure variability, ” “photoplethysmography, ” “electrodermal activity, ” “anti-G straining maneuver, ” “AGSM, ” “anti-G suit, ” “pressure breathing, ” “physiological monitoring, ” “multimodal data fusion, ” and “G-LOC prediction.” Related terms such as “orthostatic stress, ” “lower-body negative pressure, ” “hypoxia, ” “cerebral hypoperfusion, ” “neural connectivity, ” and “loss of consciousness” were also used when the evidence addressed mechanisms relevant to G-LOC.

No strict date restriction was applied to foundational studies on G-LOC physiology, +Gz tolerance, cerebral perfusion, and anti-G countermeasures because several historically important studies remain central to the field. For recent topics such as wearable physiological monitoring, multimodal data fusion, machine-learning-based prediction, and adaptive protection technologies, greater emphasis was placed on contemporary studies published within the last decade and on the most recent available literature up to the final revision of this manuscript.

### 2.3 Inclusion and exclusion criteria

Studies were considered eligible when they met one or more of the following criteria: (1) they directly investigated +Gz exposure, G tolerance, A-LOC, G-LOC, visual symptoms, cognitive impairment, or recovery after high-G exposure; (2) they examined cardiovascular regulation, cerebral perfusion, cerebral oxygenation, neural activity, respiratory mechanics, or behavioral performance under +Gz or mechanistically comparable physiological stress; (3) they evaluated anti-G countermeasures, including AGSM, anti-G suits, pressure breathing, centrifuge training, adaptive training, or human–machine protective systems; or (4) they reported physiological monitoring, signal-processing, multimodal fusion, or prediction methods with potential relevance to G-LOC risk assessment.

Studies were excluded when they were not relevant to +Gz exposure, cerebral hypoperfusion, physiological monitoring, or operational impairment; when they focused on unrelated clinical loss-of-consciousness mechanisms without transferable physiological relevance; or when they did not provide sufficient methodological or conceptual detail to support interpretation. Evidence from adjacent fields, including orthostatic stress, lower-body negative pressure, hypoxia, altered-gravity research, anesthesia-related consciousness studies, and multimodal biomedical signal processing, was included only when it addressed mechanisms or methods that were explicitly relevant to G-LOC interpretation. Such evidence was treated as supportive rather than as direct evidence for operational high-G incapacitation.

### 2.4 Assessment of relevance and evidence quality

Because this is a narrative review, no formal risk-of-bias scoring or quantitative evidence grading was performed. Instead, selected studies were evaluated according to their relevance to G-LOC mechanisms and operational high-G exposure. Greater weight was assigned to human centrifuge studies, in-flight or aircrew studies, and studies reporting objective physiological or behavioral outcomes under +Gz exposure. Studies involving surrogate conditions, animal models, computational simulations, or adjacent consciousness research were used primarily to clarify mechanisms, generate hypotheses, or identify future research directions.

The quality and relevance of the evidence were judged using the following criteria: directness of the relationship to G-LOC or +Gz physiology; clarity of the exposure condition and outcome definition; use of objective physiological, neural, behavioral, or operational measures; methodological transparency; consistency with established cardiovascular and cerebral perfusion physiology; and applicability to high-performance aviation. On this basis, the review distinguishes between established physiological pathways, candidate monitoring indicators, and emerging or hypothetical mechanisms requiring further validation.

## 3 Physiological mechanisms of G-LOC

**Figure 1:**
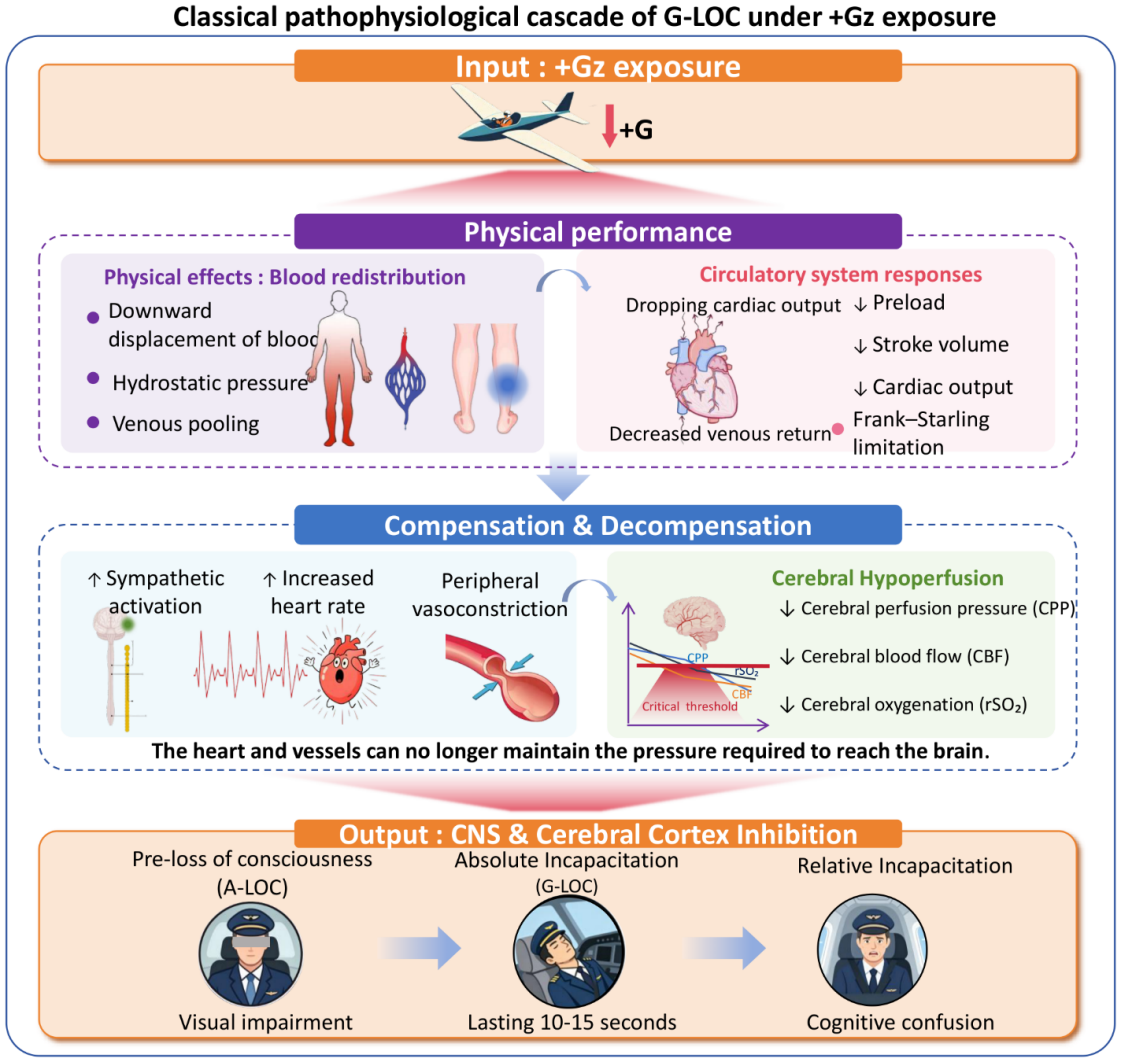
Classical pathophysiological cascade of G-LOC under +Gz exposure. +Gz exposure promotes lower-body blood pooling, reducing venous return, cardiac output, cerebral perfusion pressure, and cerebral oxygenation. When compensatory cardiovascular responses are insufficient, progressive neural and functional impairment may occur, ranging from visual disturbance and A-LOC to complete G-LOC and delayed recovery.

G-LOC arises from the inability of the cardiovascular system to maintain adequate cerebral perfusion under increased +Gz acceleration. Rather than a single-cause phenomenon, it reflects the coupled dynamics of circulatory regulation, cerebral blood flow, and neural function (**Figure 1**).

This section follows a mechanism-oriented narrative review approach rather than a quantitative meta-analysis. The evidence synthesis followed three principles. First, studies directly involving +Gz exposure, human centrifuge experiments, A-LOC, G-LOC, pilot performance degradation, cerebral oxygenation, EEG, NIRS/fNIRS, cardiovascular regulation, and anti-G countermeasures were treated as the primary evidence base. Second, related evidence from orthostatic stress, lower-body negative pressure, hypoxia, altered-gravity studies, and anesthesia/sleep-related consciousness research was used only when it addressed mechanistically comparable processes, such as cerebral hypoperfusion, neurovascular coupling, autonomic regulation, thalamocortical disruption, or recovery of neural function. Third, mechanistic conclusions were interpreted along a causal chain linking external +Gz loading, cardiovascular compensation, cerebral perfusion pressure, cerebral oxygenation, neural network integrity, and behavioral or operational impairment (Grant and Booth, 2009; Ferrari, 2015; Baethge et al., 2019).

### 3.1 Hemodynamic changes

Under +Gz exposure, hydrostatic pressure gradients drive blood redistribution toward the lower body, particularly the lower extremities and abdominal venous compartments. This leads to venous pooling, reduced venous return, and decreased ventricular filling (Norsk, 1992; Ossard et al., 1994). As venous return declines, the Frank–Starling mechanism becomes less effective, resulting in reduced end-diastolic volume, stroke volume, and cardiac output (Tripp et al., 2006).

Compensatory cardiovascular responses are activated to maintain arterial pressure and cerebral perfusion. These include increased heart rate, enhanced sympathetic activity, and peripheral vasoconstriction. However, under high-magnitude, prolonged, or rapid-onset +Gz exposure, these responses may become insufficient. High-G exposure is associated with rapid heart-rate elevation, transient arrhythmic events, and other cardiac functional changes; animal evidence also suggests that acceleration-associated stress may contribute to myocardial injury (Hanada et al., 2004; Gül and Salmanoğlu, 2012; Öztürk et al., 2012; Zhang et al., 2015).

### 3.2 Changes in consciousness and neural function

Altered consciousness is the defining manifestation of G-LOC. A typical episode may progress from almost loss of consciousness (A-LOC) to complete incapacitation and then to a recovery phase. During A-LOC, the pilot may remain nominally conscious but show impaired attention, slowed response, reduced judgment, and degraded task execution. If cerebral perfusion and neural function continue to deteriorate, complete G-LOC occurs, during which voluntary control is lost.

After consciousness returns, residual impairment may persist. This relative incapacitation phase can include confusion, delayed responses, impaired orientation, and reduced operational performance (Shender et al., 2003). Therefore, recovery of basic consciousness does not necessarily indicate immediate restoration of functional capacity.

### 3.3 Visual-function changes

The visual system is highly sensitive to changes in cerebral perfusion; therefore, visual disturbance is often one of the earliest functional manifestations preceding G-LOC. During +Gz exposure, pilots may experience a progressive sequence of visual symptoms, including gray-out, black-out, and, in severe cases, subsequent loss of consciousness (Werchan, 1991). Gray-out is typically characterized by dimming of the visual field or reduced color perception, whereas black-out refers to complete visual loss while consciousness may still be temporarily preserved (Whinnery and Forster, 2015).

In addition to transient visual-field impairment, high-G exposure may also produce temporary effects on optic nerve function. For example, a case report described in-flight G-induced retrobulbar optic neuropathy in a fighter pilot, with gradual recovery after the event (Taylor and Chen, 2021). Although such findings should not be generalized from isolated cases, they suggest that high-G exposure may affect visual function through both perfusion-related and neuro-ophthalmic pathways.

### 3.4 Cerebral perfusion and oxygenation changes

Reduced cerebral perfusion is the central physiological pathway leading to G-LOC. During +Gz exposure, decreased cardiac output and arterial pressure at brain level reduce cerebral perfusion pressure. Studies using transcranial Doppler ultrasound and near-infrared spectroscopy have shown that cerebral blood flow velocity and cerebral oxygenation may progressively decline before G-LOC (Ryoo et al., 2002; Eiken et al., 2017). In severe cases, cerebral blood flow may approach ischemic functional thresholds or even show transient flow reversal (Sandler et al., 1977; Florence et al., 2001). Related evidence from lower-body negative-pressure studies also demonstrates coupled changes in cerebral hemodynamics and brain functional activity during central hypovolemia (Han et al., 2009).

Importantly, restoration of cerebral oxygenation after G release does not necessarily mean immediate recovery of neural function. EEG and performance-related studies suggest that neural and functional recovery may lag behind hemodynamic recovery (Tripp et al., 2006). A similar temporal dissociation has been emphasized in military-aviation hypoxia research, where recovery of brain function may lag behind arterial reoxygenation after acute hypoxic exposure (Shaw et al., 2021). This temporal dissociation highlights the need to consider both cerebral oxygenation and functional state when evaluating G-LOC progression and recovery.

### 3.5 Cognitive and memory-function changes

High-G exposure can also affect higher-order cognitive functions. Reduced cerebral perfusion and oxygenation may impair attention, reaction time, decision-making, situation awareness, and memory-related processing. Experimental evidence suggests that memory retrieval may be relatively preserved in some conditions, whereas memory encoding and the formation of new memories are more vulnerable during G exposure (Levin et al., 2007). Repeated high-G exposure may also influence learning and memory-related processes (Chen et al., 2016).

### 3.6 Network-level neural function during G-LOC progression

In addition to cerebral perfusion and oxygenation changes, G-LOC may also involve transient disruption of large-scale neural network function. Consciousness and operational performance depend on coordinated interactions among ascending arousal systems, thalamocortical circuits, frontoparietal control networks, visual-processing pathways, and sensorimotor networks. During +Gz exposure, progressive cerebral hypoperfusion and deoxygenation may weaken functional integration among these networks, thereby contributing to visual disturbance, attentional narrowing, delayed responses, impaired decision-making, and eventual loss of consciousness.

This network-level interpretation is consistent with the observation that functional impairment may occur before complete G-LOC and may persist after basic consciousness returns. Visual symptoms may reflect early vulnerability of visual-processing pathways, whereas impaired attention, reaction time, and situation awareness suggest disruption of frontoparietal and sensorimotor control networks. After G release, residual confusion and delayed operational recovery may indicate that restoration of cerebral oxygenation does not necessarily imply immediate recovery of coordinated neural function.

Direct evidence on functional or effective connectivity during high +Gz exposure remains limited because EEG, fNIRS, and other neurophysiological methods are difficult to implement under centrifuge or cockpit conditions. Nevertheless, adjacent consciousness research shows that unconsciousness is often associated with disruption of thalamocortical functional connectivity, impaired frontoparietal communication, and reduced cortical effective connectivity (Boveroux et al., 2010; Ferrarelli et al., 2010; Hudetz, 2012; Akeju et al., 2014; Mashour, 2024). Future G-LOC studies should therefore examine dynamic connectivity across compensated, decompensated, A-LOC, G-LOC, and recovery phases using EEG-based measures such as coherence, phase-locking value, phase-lag index, Granger causality, directed transfer function, and partial directed coherence, as well as fNIRS-based functional connectivity when feasible (Stam et al., 2007; Friston, 2011; Haufe et al., 2011).

## 4 Dynamic mechanistic framework of G-LOC

From a physiological perspective, the key issue is not only why G-LOC occurs, but how a pilot transitions from a compensated state to a degraded or incapacitated state under time-varying +Gz exposure. This section therefore develops a dynamic mechanistic framework of G-LOC. It focuses on the physiological basis of the transition from compensation to decompensation, the temporal mismatch underlying rapid G-LOC, and the role of individual variability in shifting the stability boundary. The translation of this mechanistic framework into observable indicators, risk-state outputs, and intervention logic is addressed in Section 5.

### 4.1 Conventional G tolerance assessment methods and their limitations

Traditionally, the assessment of G-LOC risk has relied primarily on centrifuge testing, in which an individual’s tolerance is evaluated by measuring the maximum +Gz level that can be sustained under specified conditions. Owing to its high degree of controllability and reproducibility, this approach has been widely adopted in pilot selection and training programs (Norsk, 1992; Kumar, 2023).

First, it depends on endpoint indicators such as visual impairment or loss of consciousness, which do not capture the transition from stable to unstable physiological states (Sundblad et al., 2016). Second, real flight exposure involves complex temporal profiles, including rapid onset and transient peaks, which are not adequately represented in controlled testing environments. Third, population-based thresholds neglect substantial inter-individual variability in cardiovascular regulation and physiological reserve. Consequently, maximum tolerable +Gz alone is insufficient for accurate prediction of operational risk. Therefore, conventional G-tolerance assessment provides useful standardized information, but it does not adequately characterize the time-dependent transition from physiological compensation to decompensation.

### 4.2 Time-dependent progression and rapid decompensation

During early +Gz exposure, baroreflex-mediated increases in heart rate, myocardial contractility, and peripheral vasoconstriction help maintain arterial pressure and cerebral perfusion within the individual’s compensatory reserve (Goodman et al., 2006). At moderate G loads or under gradual onset conditions, these responses may temporarily stabilize cerebral perfusion and delay neurological impairment. However, as +Gz magnitude, exposure duration, or rate of onset increases, gravitational blood pooling reduces venous return and limits ventricular filling. Consequently, cardiac output may decline despite sympathetic activation, shifting the system from compensated regulation toward circulatory decompensation (Convertino, 2001).

This transition is accompanied by progressive reductions in mean arterial pressure, cerebral perfusion pressure, cerebral blood flow, and cerebral oxygenation. As cerebral oxygen delivery becomes insufficient to sustain normal neural activity, functional impairments such as visual disturbance, slowed responses, impaired attention, and cognitive degradation may emerge before complete loss of consciousness (Van Lieshout et al., 2003). Therefore, the onset of G-LOC should be understood as a time-dependent physiological trajectory rather than as an abrupt event defined only by a fixed +Gz threshold.

Rapid-onset G-LOC represents a time-compressed form of this instability. When the rate of +Gz increase exceeds the response time of baroreflex-mediated cardiovascular adjustment and cerebrovascular regulation, cerebral perfusion pressure may fall before compensatory mechanisms are fully established (Whinnery and Forster, 2013). Under these conditions, visual warning symptoms may be shortened or absent, and the transition from apparent functional stability to incapacitation may occur within a narrow temporal window.

In addition to hemodynamic delay, rapid-onset acceleration has historically been discussed in relation to interactions among cerebral hypoperfusion, cerebrovascular regulation, and intracranial pressure or mechanical effects (Glaister, 1978). Early work on cerebral circulation during gravitational stress suggested that changes in intracranial and cerebrospinal-fluid pressure may influence the maintenance of cerebral blood flow under acceleration, leading to the concept that cerebral perfusion may transiently persist beyond what would be predicted from simple hydrostatic pressure calculations alone (Henry et al., 1951). Transcranial Doppler pulsatility index has also been reported to reflect intracranial pressure in other clinical settings, although this evidence was not obtained under high-G exposure (Bellner et al., 2004). However, this historical concept should not be interpreted as evidence that intracranial mechanical stress is a primary cause of G-LOC. Current physiological and experimental evidence continues to support reduced cerebral perfusion and insufficient cerebral oxygen delivery as the predominant mechanisms underlying G-LOC (Werchan, 1991; Kurihara et al., 2007; Whinnery and Forster, 2013). Therefore, intracranial mechanical or inertial effects, if present, should be regarded as possible secondary modifiers under rapid-onset or high-rate acceleration conditions, rather than as an independent or definitively demonstrated primary mechanism.

This temporal interpretation is important for dynamic physiological assessment. It implies that G-LOC risk may escalate before conventional tolerance limits are reached, particularly when external G loading changes faster than the pilot’s cardiovascular and cerebrovascular compensatory systems can respond. Thus, G-LOC is better conceptualized as a dynamic stability transition in which the timing, magnitude, and rate of physiological compensation determine whether the pilot remains within a stable functional state or progresses toward neural impairment and incapacitation.

### 4.3 Individual variability

Substantial inter-individual variability exists in G-LOC susceptibility under similar +Gz exposure conditions (Blue et al., 2023). This variability is mainly related to differences in autonomic regulation, cardiovascular reserve, anthropometric characteristics, flight experience, and training adaptation. Cardiopulmonary reserve may also contribute to high-G tolerance and inter-individual differences (Lan et al., 2023). Baroreflex responsiveness and vasoconstrictor reserve are particularly important because they determine how rapidly arterial pressure and cerebral perfusion can be stabilized during gravitational stress (Convertino, 2001; Sundblad et al., 2014; Sundblad et al., 2016).

Anthropometric and physical characteristics may also affect G tolerance, but their predictive value is limited when used alone. Body size and body surface area have been associated with stroke volume and orthostatic stability under acceleration stress (Nordine et al., 2015), while repeated exposure and AGSM training may improve adaptation to high-G environments (Lalande and Buick, 2009; Park et al., 2015; Eiken et al., 2025). However, single indicators such as anthropometry or aerobic capacity cannot reliably predict G tolerance across individuals (Park et al., 2015; Zeigler and Acevedo, 2024). Therefore, individual variability is best understood as an adaptive stability-boundary problem: the same +Gz profile may remain tolerable for one pilot but push another closer to physiological decompensation.

### 4.4 Integrated multi-pathway mechanistic model

**Figure 2:**
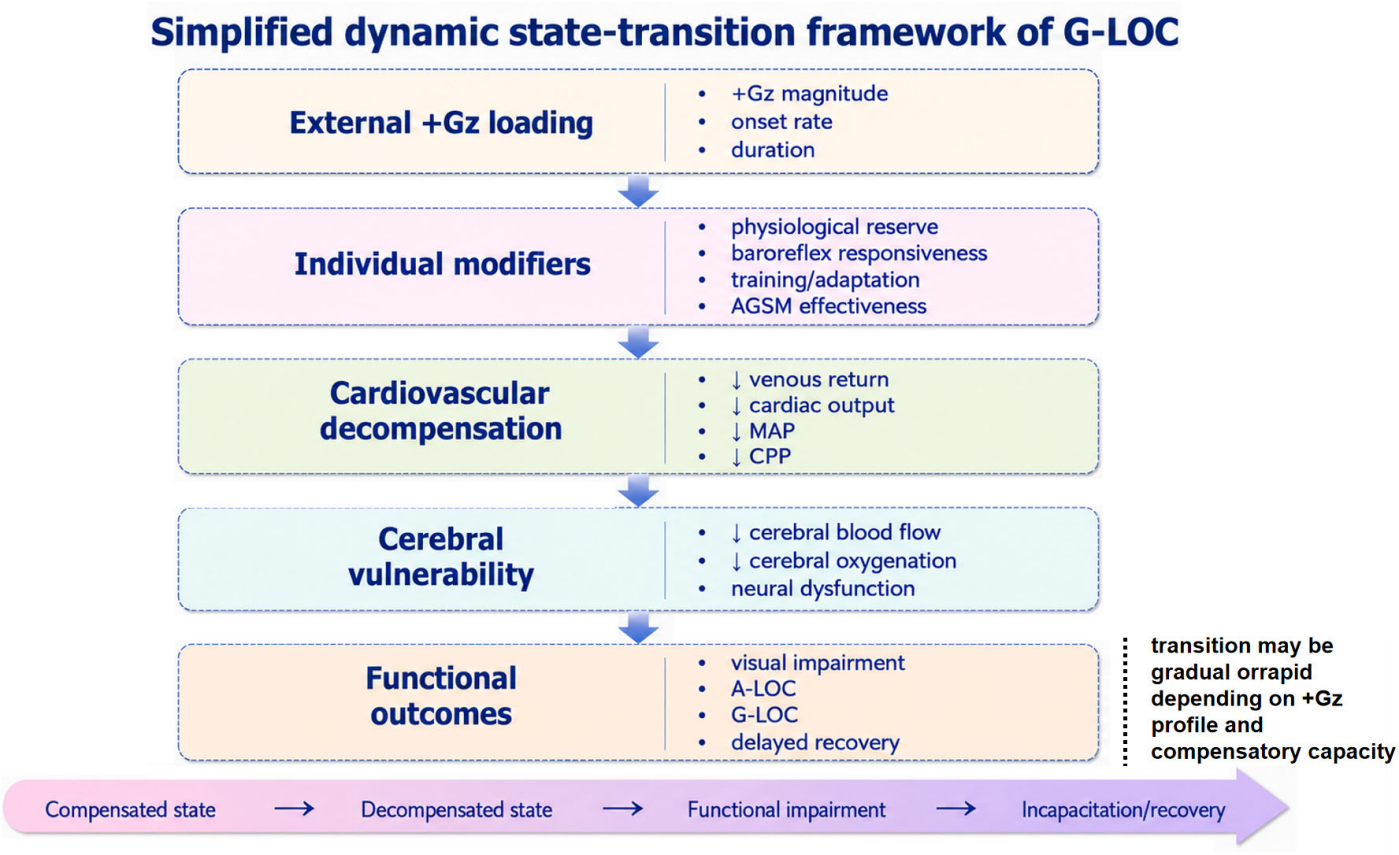
Dynamic framework of G-LOC development under +Gz exposure. The image summarizes G-LOC as a dynamic transition from compensated regulation to cardiovascular decompensation, cerebral vulnerability, functional impairment, and incapacitation or recovery. External +Gz loading, defined by magnitude, onset rate, and duration, interacts with individual modifiers such as physiological reserve, baroreflex responsiveness, training/adaptation, and AGSM effectiveness. When compensatory capacity is exceeded, reduced venous return, cardiac output, MAP, and CPP may lead to decreased cerebral blood flow, reduced cerebral oxygenation, neural dysfunction, visual impairment, A-LOC, G-LOC, and delayed recovery. The transition may occur gradually or rapidly depending on the +Gz profile and compensatory capacity.

Based on the above evidence, G-LOC can be conceptualized as a dynamic stability transition in a coupled physiological system rather than as a fixed-threshold event (**Figure 2**). Within this framework, three classes of variables interact:

1. External inputs: +Gz magnitude, rate of onset, and duration
2. Circulatory dynamics: venous return, cardiac output, MAP, and CPP
3. Modulatory factors: individual physiological capacity and regulatory time constants

As +Gz increases, reduced venous return constrains cardiac output, while baroreflex mechanisms attempt to maintain MAP and CPP. When compensatory capacity is sufficient, the system remains stable. However, under high load or rapid onset, the system may cross a critical tipping point, leading to rapid decline in CPP and loss of neural function.

Compared with conventional G-tolerance models, which primarily describe tolerance as a maximum sustainable +Gz level, a time-to-endpoint relationship, or the occurrence of visual symptoms and loss of consciousness, the present framework advances the interpretation of G-LOC in three respects. First, it shifts the analytical focus from endpoint tolerance to the dynamic trajectory of physiological state before, during, and after overt incapacitation. This allows G-LOC risk to be understood as a progression from compensated cardiovascular regulation to cerebral vulnerability, functional degradation, A-LOC, complete G-LOC, and delayed recovery. Second, it integrates multiple interacting pathways, including external G-load characteristics, circulatory regulation, compensatory time constants, cerebral perfusion and oxygenation, neural functional integrity, individual physiological reserve, active countermeasure execution, and operational performance. In this sense, the framework does not replace classical cerebral hypoperfusion theory; rather, it extends it by embedding cerebral hypoperfusion within a broader time-dependent and multi-system physiological process. Third, it provides a translational bridge between classical G-LOC physiology and future monitoring-guided protection systems by linking mechanistic variables to candidate physiological indicators, individualized state assessment, AGSM feedback, adaptive anti-G protection, and human–machine intervention.

Accordingly, this framework should be interpreted as an integrative synthesis and operationally oriented conceptual framework rather than as a newly validated theoretical model. Its value lies in organizing existing evidence into a structure that can guide future research on when physiological compensation begins to fail, which signals may provide early warning, and how protective interventions might be timed before complete incapacitation occurs. However, the framework itself does not determine which observable signals can reliably identify the transition from compensated regulation to functional impairment. The next section therefore translates this mechanistic understanding into candidate physiological and behavioral indicators, state-estimation principles, and adaptive protection strategies for high-G aviation environments.

## 5 Physiological monitoring and leading indicators of G-LOC progression

Building on the dynamic physiological framework described above, physiological monitoring for G-LOC should focus on observable signals that may capture the transition from compensated cardiovascular and cerebrovascular regulation to functional impairment. These signals can be organized into five monitoring domains: cardiovascular-autonomic and peripheral perfusion indicators, including HR, HRV, arterial pressure, BPV, thoracic impedance, PPG-derived waveform features, arrhythmic events, CFI, and EDA; cerebral oxygenation and neural indicators, including EEG and NIRS/fNIRS-derived oxygenation kinetics; respiratory, ventilation, and oxygenation-related indicators, including respiratory pattern, respiratory mechanics, SpO_2_, and EIT-derived ventilation distribution; electromyographic indicators of AGSM execution; and behavioral or task-performance indicators. These domains should not be interpreted as isolated physiological systems, but as complementary observation windows on the progression from circulatory compensation to cerebral vulnerability and functional degradation (Shaw and Harrell, 2023). Their characteristics, advantages, limitations, and evidence types are summarized in **Table 2**.

**Table 2:** Physiological and behavioral indicators for G-LOC monitoring.

Physiological signal	Measurement technique	Relevance to G-LOC	Evidence type	Physiological indicator	Advantages	Limitations	Representative references
Electrocardiogram (ECG)	Wearable electrodes	High	Candidate/supportive indicator	Heart rate (HR), heart rate variability (HRV)	Mature technology, high temporal resolution	Motion artifacts; indirect indicator of cerebral perfusion	Green and Ford, 2006; Lyons et al., 2004
Blood pressure	Finger cuff or arterial sensors	High	Established pathophysiological indicator	Mean arterial pressure (MAP)	Direct measurement of circulatory status	Sensor complexity; calibration required	Lyons et al., 2004; Sevilla and Gardner, 2005
Blood pressure variability (BPV)	Beat-to-beat blood pressure monitoring, finger cuff, or arterial pressure sensors	High	Candidate/supportive indicator	Short-term BPV, beat-to-beat pressure instability, pressure recovery dynamics	Reflects temporal stability of arterial pressure regulation and baroreflex buffering; may provide information beyond absolute MAP	Requires reliable beat-to-beat pressure measurement; sensitive to motion, AGSM, sensor artifacts, and analysis-window selection	Parati et al., 1995; Convertino, 2001; Sundblad et al., 2016
Cardiac force index (CFI)	Mobile device or wearable-derived body weight, activity, and heart-rate parameters	Supportive	Experimentally tested susceptibility indicator.	CFI or wearable-derived cardiovascular reserve index	Useful for individualized baseline assessment of G tolerance and cardiovascular reserve	Not a direct marker of acute cerebral hypoperfusion; more suitable for pre-exposure susceptibility assessment than real-time G-LOC confirmation	Kuo et al., 2023
Photoplethysmography (PPG)	Optical finger or ear sensors	Indirect	Candidate/supportive indicator	Pulse wave and peripheral perfusion	Compact, wearable, low power consumption	Sensitive to motion and peripheral vasoconstriction	Kim and Koh, 2025; Green and Ford, 2006; Sammito et al., 2026
Electrodermal activity (EDA)	Skin conductance electrodes, usually on fingers, palm, wrist, or wearable devices	Supportive	Candidate/supportive indicator	Skin conductance level, skin conductance response, sympathetic arousal	Provides complementary information on autonomic arousal, stress, workload, and sympathetic activation	Not a direct marker of cerebral hypoperfusion or loss of consciousness; sensitive to sweating, skin temperature, emotional state, electrode contact, and cockpit environment	Boucsein, 2012; Yu et al., 2024
Electroencephalography (EEG)	Scalp electrodes	Very High	Established pathophysiological indicator	Cortical electrical activity	Direct indicator of neural function and consciousness	Sensitive to noise; difficult to implement in operational cockpit	Whinnery and Whinnery, 1990; Blacker and Dooley, 2024
Near-infrared spectroscopy/functional NIRS (NIRS/fNIRS)	Optical sensors on forehead or wearable fNIRS system	Very high	Established pathophysiological indicator	Cerebral oxygenation, Oxy-Hb, Deoxy-Hb, deoxygenation slope, reoxygenation slope, recovery delay	Non-invasive monitoring of cerebral oxygenation; closer to the downstream pathway of G-LOC than peripheral indicators	Limited penetration depth; sensitive to superficial blood flow, motion, sensor displacement, helmet integration, and inter-individual anatomical variability	Kurihara et al., 2007; Sharma et al., 2021; Kim and Koh, 2025; Roumengous et al., 2024
Ventilation distribution/electrical impedance tomography (EIT)	Thoracic EIT electrode belt or impedance imaging system	Research-use /indirect	Candidate/supportive indicator	Regional ventilation distribution, gravity-induced ventilation shift, lung impedance change	Provides spatial information on lung mechanics and ventilation distribution under high-G acceleration	More suitable for centrifuge or laboratory studies; limited cockpit feasibility due to electrode placement, hardware integration, motion sensitivity, and operational complexity	Menden et al., 2021
Respiratory signals	Chest band, airflow sensor, thoracic impedance, or respiratory inductance plethysmography	Indirect	Candidate/supportive indicator	Respiratory rate, respiratory effort, breathing phase, respiratory coordination during AGSM	Useful for assessing AGSM performance and respiratory contribution to oxygen transport	Limited direct relationship with cerebral perfusion; affected by workload, equipment constraints, and breathing strategy	Whinnery and Whinnery, 1990; Lyons et al., 2004; Tu et al., 2020; Pollock et al., 2021
Electromyography (EMG)	Surface electrodes	Indirect	xperimentally tested candidate predictor	Muscle activation	Reflects execution quality of anti-G straining maneuver; relatively easy to integrate	Indirect measure; sensitive to motion artifacts; muscle fatigue effects	Choi et al., 2015; Kim et al., 2017
Behavioral/performance indicators	Flight control input, eye tracking, task performance metrics	Supportive	Candidate/supportive indicator	Reaction time, control variability, gaze behavior	Directly reflects operational performance; relevant to real-world task	Not physiological; influenced by task context; requires integration with other signals	Parasuraman et al., 2000; Wilson and Russell, 2007

Cardiovascular and peripheral perfusion indicators reflect systemic circulatory regulation, autonomic compensation, peripheral perfusion, and cardiovascular reserve under +Gz exposure. ECG and heart-rate-related measures are technically mature and provide high temporal resolution, making them suitable for continuous monitoring (Blue and Ong, 2024). Arterial pressure is more directly related to circulatory stability, whereas BPV may provide additional information on the temporal stability of pressure regulation and baroreflex buffering. Increased short-term BPV or unstable beat-to-beat pressure responses may indicate impaired cardiovascular compensation under acceleration stress (Convertino, 2001; Sundblad et al., 2016; Parati et al., 1995). Thoracic impedance and PPG-derived pulse morphology, including pulse amplitude attenuation, pulse wave flattening, pulse arrival time, and pulse transit time, may further reflect changes in central blood volume, peripheral perfusion, and cuff-free blood-pressure estimation (Hanada et al., 2004; Howarth et al., 2008; Kviesis-Kipge and Rubins, 2016; Sarkar and Pahuja, 2024; Sammito et al., 2026). However, these peripheral indicators are sensitive to motion artifacts, sensor contact pressure, vasoconstriction, temperature, and AGSM-related muscle activity. They should therefore be interpreted together with cerebral oxygenation, arterial pressure, EMG, and behavioral indicators rather than as standalone evidence of imminent G-LOC. CFI may support individualized baseline assessment of cardiovascular reserve and G tolerance, but it is more appropriate for pre-exposure susceptibility assessment than for real-time confirmation of cerebral hypoperfusion (Kuo et al., 2023).

Cerebral oxygenation and neural-function measures are closer to the final physiological pathway of G-LOC. EEG reflects cortical electrical activity and is directly related to consciousness-related neural function (Wilson et al., 2005; Blacker and Dooley, 2024), whereas NIRS/fNIRS-derived cerebral oxygenation provides a non-invasive measure of cerebral oxygen supply during +Gz exposure (Li et al., 2010; Roumengous et al., 2024). Compared with a single oxygenation value, dynamic features such as the rate of Oxy-Hb decrease, Deoxy-Hb increase, deoxygenation slope, reoxygenation slope, and recovery delay may better describe the temporal coupling among +Gz exposure, cerebral perfusion compromise, and neural vulnerability (Benni et al., 2003b; Ryoo et al., 2004; Kurihara et al., 2007; Roumengous et al., 2024). Threshold-based models linking cerebral perfusion interruption to functional impairment further support the mechanistic relevance of these variables (Moore et al., 1993). Nevertheless, cerebral indicators remain constrained by inter-individual variability, extracerebral blood-flow contamination, motion artifacts, sensor displacement, helmet integration, and temporal inconsistencies between neural and oxygenation signals (Kurihara et al., 2007; Iwasaki et al., 2012). Therefore, fNIRS and EEG should be interpreted as components of a multimodal physiological trajectory rather than as isolated endpoints.

Respiratory, EDA, EMG, and behavioral indicators provide complementary information about oxygen transport, autonomic arousal, countermeasure execution, and functional degradation. Respiratory patterns are relevant because effective AGSM requires coordinated forced respiration, thoracic pressure regulation, rapid air exchange, and lower-body muscle straining to support arterial pressure and cerebral perfusion (Yang et al., 2007; Tu et al., 2020; Junior et al., 2024). Respiratory mechanics, thoracic impedance, and EIT-derived ventilation distribution may further clarify how high-G exposure alters lung mechanics, regional ventilation, and gas exchange, although EIT is currently more suitable for centrifuge or laboratory studies than routine cockpit deployment (Menden et al., 2021; Pollock et al., 2021). EDA may help characterize sympathetic arousal, workload, stress, and autonomic imbalance during acceleration exposure, but it should not be interpreted as a direct marker of cerebral hypoperfusion or loss of consciousness (Boucsein, 2012; Yu et al., 2024). EMG extends monitoring from passive physiological observation to evaluation of active AGSM execution. Lower-limb EMG, especially from the gastrocnemius, can indicate whether the pilot is successfully engaging a critical countermeasure strategy (Choi et al., 2015; Kim et al., 2017). Behavioral and task-performance indicators, including reaction time, control-input stability, tracking error, gaze behavior, posture, and task-performance metrics, reflect the functional consequences of physiological degradation and are operationally relevant because impairment may occur before complete loss of consciousness (Tripp et al., 2006; Shender et al., 2003; Rickards and Newman, 2005). However, these indicators are affected by task demands, workload, fatigue, training level, cockpit interface, and operational context, and should therefore be interpreted together with physiological signals rather than as independent markers (Wingelaar-Jagt et al., 2021).

Although these indicators are mechanistically relevant, their predictive validity differs substantially. They can be divided into three evidence categories: established pathophysiological indicators, experimentally tested predictors, and candidate leading indicators. Cerebral oxygenation and neural-function measures are established pathophysiological indicators because they reflect downstream cerebral perfusion and consciousness-related neural activity. However, strong mechanistic relevance does not necessarily mean operational predictive validation. Thresholds for cerebral oxygenation decline vary across individuals, G profiles, sensor placement, instrumentation, and analysis methods. Most available studies were conducted in centrifuge settings and were not designed to provide prospective sensitivity, specificity, false-alarm rate, or warning lead-time estimates under operational flight conditions.

Only a limited number of studies have reported quantitative predictive performance for G-LOC or G-tolerance-related outcomes. A logistic-regression formula using body mass index and the rate of change from maximum to minimum cerebral oxyhemoglobin reported a sensitivity of 67.6%, specificity of 81.4%, and accuracy of 79.5% for G-LOC prediction in centrifuge trainees (Ohrui et al., 2017). However, because this approach depends on the maximum-to-minimum oxyhemoglobin change during +Gz exposure, it is better interpreted as a *post hoc* or near-endpoint prediction method than as a real-time early-warning model with a clearly established lead time. More recently, an SVM-based model using age, height, weight, anti-G suit status, +Gz level, cerebral oxyhemoglobin, and deoxyhemoglobin was tested in 124 flight-course students during centrifuge training. The Gaussian-kernel SVM achieved approximately 63.7–65.3% accuracy after 2.5 s from the onset of high +Gz exposure, with precision of 62.7–65.1% and recall of 62.9–71.0% (Ohrui et al., 2024). These findings provide useful proof-of-concept evidence, but the reported performance remains insufficient for operational deployment, especially where missed detections or false alarms could trigger inappropriate warnings or automated recovery actions.

Other signals should currently be treated as candidate leading indicators rather than validated operational predictors. EMG features from the gastrocnemius muscle have shown potential warning value, with rapid decreases in waveform length, integrated absolute value, root mean square, and mean absolute value during the alarm phase approximately 3 s before G-LOC (Choi et al., 2015; Kim et al., 2017). However, complete sensitivity, specificity, diagnostic accuracy, and external validation under operational flight conditions remain insufficient. Similarly, HR, HRV, BPV, PPG waveform morphology, EDA, respiratory variables, and behavioral performance measures may provide physiologically meaningful information about cardiovascular regulation, autonomic arousal, peripheral perfusion, AGSM coordination, and functional degradation, but G-LOC-specific predictive metrics are generally unavailable or insufficiently validated. CFI and HRV may be useful for pre-exposure assessment of G tolerance or cardiovascular reserve, but they should not be described as real-time predictors of imminent G-LOC unless validated against synchronized +Gz exposure, cerebral oxygenation, behavioral impairment, and G-LOC outcomes (Kuo et al., 2023; Bacevic et al., 2024).

A further limitation concerns the translation from centrifuge-based evidence to operational flight. Centrifuge experiments provide controlled +Gz profiles, standardized monitoring, and clearer outcome labeling, which are essential for model development. However, they do not fully reproduce the combined effects of real flight, including dynamic maneuver sequences, cockpit vibration, sensor displacement, pressure breathing, workload, fatigue, dehydration, hypoxia, mission stress, decision-making demands, and aircraft-control consequences. Operational flight data are more ecologically valid but difficult to collect, rarely include true G-LOC events, and often lack synchronized multimodal physiological, behavioral, and flight-state measurements. Therefore, future studies should report sensitivity, specificity, diagnostic accuracy, precision, recall, false-alarm burden, missed-detection risk, temporal lead time before functional impairment or G-LOC, robustness to artifacts and missing modalities, and cross-subject, cross-device, and cross-scenario validation. Until such evidence is available, most physiological variables should be described as promising candidate indicators rather than validated operational predictors.

## 6 Mechanism-informed countermeasures and adaptive physiological protection

The preceding section defined how physiological and behavioral indicators can be translated into risk states and intervention triggers. This section focuses on the technologies required to implement that logic in practice, including sensing, fusion, prediction, protective actuation, training, and human–machine integration.

Existing G-LOC protection technologies can be organized into four functional layers: baseline protection, real-time monitoring, predictive assessment, and adaptive intervention. Baseline protection includes conventional anti-G equipment, anti-G straining maneuver (AGSM), and training-based adaptation. Real-time monitoring technologies acquire physiological, behavioral, and flight-state information. Predictive models transform these data into individualized risk outputs. Adaptive intervention technologies then use these outputs to support warnings, AGSM feedback, smart anti-G protection, workload management, or flight-control assistance. This section reviews these implementation technologies and clarifies how they contribute to dynamic G-LOC risk prevention.

### 6.1 Conventional countermeasures as baseline protection

Over decades of aerospace medicine research and engineering practice, conventional countermeasures against G-LOC have evolved into a multi-layered protection system. These approaches include mechanical anti-G equipment, active physiological techniques, and training-based adaptation. They remain the foundation of G-LOC prevention because they directly target the primary physiological pathway of +Gz-induced blood redistribution and reduced cerebral perfusion.

Mechanical protection, primarily in the form of anti-G equipment, remains the most widely implemented engineering solution. By mitigating blood pooling in the lower extremities, such systems help maintain cerebral perfusion pressure and support cardiovascular stability under +Gz stress (Whinnery et al., 2014). However, conventional anti-G equipment is largely passive or preprogrammed. Its protective effect depends mainly on G-load-triggered inflation rather than individualized physiological state. As a result, it may be insufficient under rapid-onset +Gz profiles or in pilots whose physiological reserve is temporarily reduced.

Active physiological countermeasures, particularly the anti-G straining maneuver (AGSM), directly augment circulatory compensation through coordinated muscle contraction and breathing regulation. Effective execution has been shown to significantly improve G tolerance and delay functional impairment (Whinnery et al., 2014; Pollock et al., 2024). Nevertheless, performance is highly dependent on pilot proficiency and may deteriorate under high workload or operational complexity.

Training and long-term physiological adaptation further contribute to individual variability in G tolerance. Repeated high-G exposure and targeted physical conditioning can enhance compensatory capacity, potentially through vascular and autonomic adaptations (Eiken et al., 2025; Bruno, 2026). However, these effects are gradual and cannot address acute physiological decompensation during flight.

### 6.2 Multimodal data fusion and predictive modeling

Multimodal modeling provides the computational basis for transforming physiological monitoring into individualized G-LOC protection strategies. A practical pipeline should include synchronized acquisition of flight-state, cardiovascular, cerebral, respiratory, EMG, autonomic, and behavioral data, followed by artifact rejection, temporal feature extraction, individualized baseline correction, and multimodal fusion. Because G-LOC develops through cross-system interactions among +Gz loading, cardiovascular compensation, cerebral perfusion, neural function, AGSM execution, and task performance, single-signal monitoring is unlikely to provide robust prediction across pilots and operating conditions.

Existing fusion strategies can be broadly divided into data-level, feature-level, decision-level, and hybrid fusion (Chaabene et al., 2025). Data-level fusion directly combines synchronized signal streams, but it is vulnerable to differences in sampling rate, signal quality, and motion artifacts. Feature-level fusion combines extracted variables such as HRV, BPV, PPG waveform morphology, fNIRS oxygenation kinetics, respiratory features, EDA, EMG activation, and behavioral metrics, and is useful for maintaining physiological interpretability. Decision-level fusion combines outputs from modality-specific models and may be more robust when one or more sensors are unreliable. Hybrid fusion, including attention-based, graph-based, and deep representation-learning approaches, may better capture cross-modal interactions, but it requires larger datasets and stronger validation than conventional feature- or decision-level fusion (Tejedor et al., 2019; Stahlschmidt et al., 2022; Alqudah and Moussavi, 2025).

For G-LOC risk assessment, multimodal fusion is physiologically justified. Studies integrating cardiovascular, cerebral, and autonomic indicators suggest that combined features can improve assessment of G tolerance and early warning capability (Li et al., 2014; de Sá et al., 2023; Metin et al., 2023; Kuo et al., 2023). The inclusion of BPV, PPG waveform morphology, EDA, respiratory mechanics, and fNIRS oxygenation kinetics may further improve the physiological interpretability of state estimation, provided that these features are validated against synchronized G-load and functional outcome data.

The development of prediction models has progressed from static tolerance assessment toward dynamic individualized state estimation. Traditional approaches relied mainly on maximum +Gz tolerance, exposure duration, visual symptoms, or endpoint loss of consciousness during centrifuge testing. More recent studies have incorporated physiological markers and machine-learning methods. HRV and autonomic indices have been used to evaluate +Gz tolerance and cardiovascular regulation (de Sá et al., 2023; Metin et al., 2023; Bacevic et al., 2024). Wearable-derived CFI has been proposed for individualized prediction of G tolerance and cardiovascular reserve (Kuo et al., 2023). EMG-based warning algorithms have been used to characterize AGSM-related muscle activation and G-LOC risk (Kim et al., 2017). Support vector machines, convolutional neural networks, and graph-based models have also been explored for G-LOC prediction and state recognition (Ohrui et al., 2024; Chen et al., 2024; Zhang et al., 2026). These studies indicate a clear shift from population-level thresholds toward personalized, sensor-based risk prediction.

Computational physiological models provide another route for risk prediction. By simulating cardiovascular dynamics, cerebral perfusion, and oxygenation under different G-load profiles, model-based approaches can estimate time-dependent changes in cerebral blood flow and functional impairment (McKinley et al., 2005; McKinley and Gallimore, 2013; Copeland and Whinnery, 2023). These approaches are valuable because they incorporate mechanistic knowledge and may improve interpretability. However, purely mechanistic models may be limited by simplified assumptions regarding vascular regulation, individual reserve, AGSM execution, and neurovascular coupling. In contrast, machine-learning models can learn nonlinear patterns from high-dimensional monitoring data, but they require sufficient training data and rigorous validation.

Recent gravity-awareness modeling studies have also used deep learning and large language models to simulate neurophysiological and subjective-state adaptation under altered gravitational environments. Such studies are relevant to the broader trend of computational human-state modeling because they integrate EEG, HRV, EDA, motor behavior, and generative cognitive simulation (Alibekov et al., 2026). However, they are not specific to +Gz-induced G-LOC and should not be regarded as direct physiological evidence for high-G incapacitation. For G-LOC research, their main value lies in hypothesis generation, multimodal state representation, and simulation-assisted training design. Operational use will still require validation against centrifuge or in-flight datasets containing synchronized G-load, cardiovascular, cerebral, respiratory, autonomic, behavioral, and recovery-phase measurements.

Several methodological limitations remain. First, available datasets are often small, single-center, and dominated by centrifuge conditions, whereas real-flight physiological datasets with synchronized G-load and task-performance outcomes remain scarce (Whinnery and Whinnery, 1990). Second, true G-LOC events are rare and ethically constrained, leading to class imbalance and uncertain labeling between compensated strain, visual impairment, A-LOC, G-LOC, and recovery (Whinnery and Forster, 2013). Third, many models are validated within the same experimental environment, limiting evidence for cross-subject, cross-device, and cross-scenario generalizability (Stahlschmidt et al., 2022; Zhao et al., 2024). Fourth, high +Gz exposure, AGSM, helmet movement, pressure breathing, and cockpit workload can introduce severe artifacts into EEG, fNIRS, PPG, EDA, and EMG signals (Naseer and Hong, 2015). Fifth, different modalities have different temporal dynamics: arterial pressure and ECG change rapidly, EDA changes more slowly, fNIRS has hemodynamic delay, EMG reflects active countermeasure execution, and behavioral indicators represent downstream functional consequences (Naseer and Hong, 2015). Therefore, simple feature concatenation may obscure causal timing and cross-modal dependencies (Baltrušaitis et al., 2019).

Future models should move beyond offline classification accuracy and be evaluated according to operationally meaningful criteria, including warning lead time, missed-detection risk, false-alarm burden, robustness to missing modalities, uncertainty calibration, and recovery-state assessment. Individualized modeling remains essential because the same physiological value may have different risk implications across pilots. Personalized approaches using subject-specific baselines, longitudinal records, prior G-response patterns, and adaptive calibration may improve robustness and reduce false alarms (Kantharaju et al., 2023; Ding et al., 2025; Perz and Kazienko, 2025; Paniagua-Gómez and Fernandez-Carmona, 2025; Paul et al., 2025). Ultimately, multimodal prediction should be validated not only by its ability to identify G-LOC risk, but also by whether it can support timely and safe interventions, including AGSM feedback, adaptive anti-G protection, pressure-breathing support, workload reduction, and flight-control assistance.

### 6.3 Adaptive training and AGSM feedback technologies

Training technologies improve both physiological tolerance and the quality of pilot-initiated countermeasures. Dynamic human centrifuges allow task-relevant assessment of G tolerance, AGSM timing, and recovery dynamics under realistic G-load profiles (Dančuo et al., 2012; Winter et al., 2023). Compared with simple stepwise or fixed-profile testing, dynamic exposure protocols are more suitable for evaluating the physiological responses of pilots under complex G-load conditions.

The integration of virtual reality and flight simulation with centrifuge systems further extends training from physiological tolerance to task-relevant performance. By embedding visual, cognitive, and motor tasks within high-G exposure, these systems allow simultaneous training of perception, decision-making, motor control, and AGSM execution under more realistic operational conditions (Clément and Ngo-Anh, 2013; Kim et al., 2025; Fuentes-García et al., 2025).

AI-assisted training systems can provide individualized feedback by analyzing physiological and behavioral data during training. Such systems may evaluate whether AGSM is initiated at the appropriate time, whether muscle contraction is sufficient, whether breathing is synchronized, and whether physiological recovery is delayed. Based on these data, training intensity, exposure profiles, and feedback strategies can be adapted to the individual pilot (Tao et al., 2022; Wochyński, 2025).

Thus, adaptive training technologies contribute to prevention by improving baseline tolerance and by generating individualized data for subsequent monitoring and protection.

### 6.4 Adaptive anti-G protection and pressure-breathing systems

Anti-G protection technologies are evolving from passive mechanical support toward adaptive physiological assistance. Modern anti-G suits improve protection through optimized bladder configuration, faster inflation dynamics, and improved pressure distribution. These developments enhance the ability to reduce blood pooling and support cerebral perfusion, especially under high or rapidly changing +Gz exposure (Eiken et al., 2007; Whinnery et al., 2014).

Pressure breathing for G provides additional support by increasing intrathoracic pressure and helping maintain arterial pressure and cerebral perfusion during high-G exposure (Copeland and Whinnery, 2023). When combined with anti-G suits and AGSM, pressure breathing can contribute to a more integrated physiological protection strategy.

Smart anti-G systems represent a further step toward individualized and context-aware protection (Kim and Koh, 2025). By incorporating real-time physiological data and flight-state information, such systems can dynamically adjust pressure output, inflation timing, and pressure distribution according to the pilot’s current risk state (Liu et al., 2024; Ma et al., 2025; Kim et al., 2025). This adaptive capability is particularly relevant for rapid-onset +Gz exposure, where conventional G-triggered inflation may not fully match the time course of physiological decompensation.

In a dynamic prevention architecture, adaptive anti-G protection can serve as an execution layer that translates assessment outputs into physiological support. By adjusting inflation timing, pressure distribution, and pressure-breathing support, these systems may better match the temporal profile of G-induced physiological decompensation.

### 6.5 Human–machine integrated countermeasures

Human–machine integrated intervention represents the system-level implementation of dynamic G-LOC prevention. It links physiological monitoring, predictive modeling, protective equipment, and aircraft control functions to support graded intervention. The objective is not to replace the pilot, but to maintain functional capacity and prevent transient physiological degradation from progressing into loss of aircraft control.

A human–machine integrated G-LOC protection system can be organized into three layers: multimodal sensing, intelligent assessment, and graded intervention. The sensing layer acquires physiological, behavioral, and flight-state data; the assessment layer estimates the pilot’s current risk state using physiological, machine-learning, or hybrid models; and the intervention layer implements responses according to risk severity.

Graded intervention is essential because excessive automation or unnecessary interruption may increase workload or reduce pilot trust. In a normal state, the system may continue monitoring without intervention. In an early-warning state, it may provide feedback to improve AGSM execution or increase pilot awareness. In a high-risk state, it may reduce workload, support task reallocation, adjust anti-G suit inflation, or enhance flight-control assistance. In an imminent-incapacitation state, more assertive actions such as G-load reduction, flight-envelope protection, temporary stabilization control, or automatic recovery support may be required (Suplisson, 2015; Tripp, 2007).

Human–machine integration also supports proactive workload management. By combining physiological state estimation with task demands, the system can dynamically adjust operational complexity or allocate functions between the pilot and automation (Parasuraman et al., 2000; Wilson and Russell, 2003; Wilson and Russell, 2007). This is particularly important because G-LOC risk involves not only physiological failure but also degradation of perception, cognition, and motor control. Adaptive automation can therefore reduce accident risk by intervening before complete incapacitation occurs.

**Figure 3:**
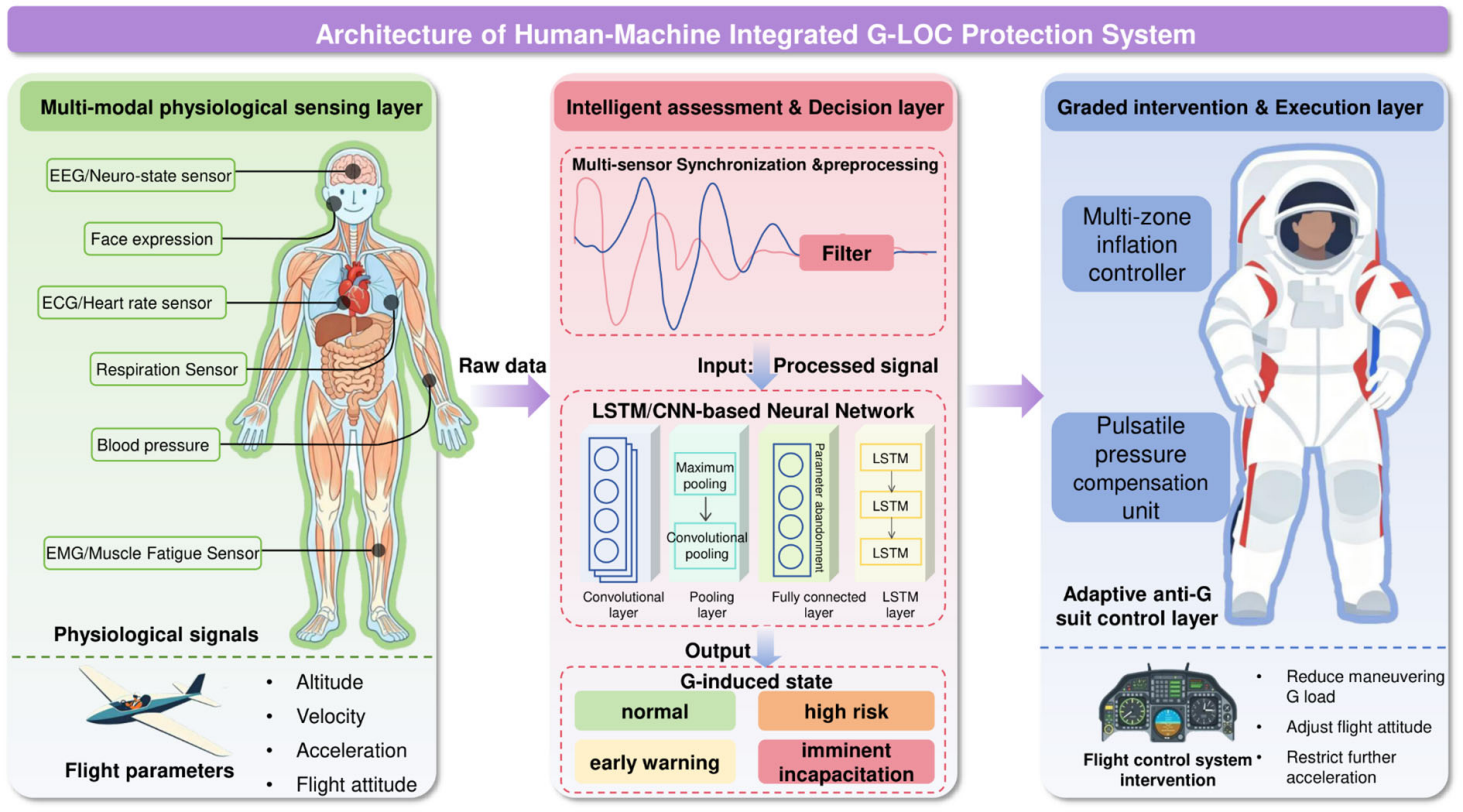
Architecture of human-machine integrated G-LOC protection system. The system comprises three layers: multimodal physiological sensing, intelligent assessment, and graded intervention. The sensing layer acquires physiological signals, behavioral indicators, and flight-state information. The assessment layer performs artifact rejection, feature extraction, data fusion, individualized modeling, and risk-state estimation. The intervention layer provides graded responses, including warning, AGSM feedback, adaptive anti-G protection, workload management, and flight-control assistance.

**Figure 3** illustrates this implementation architecture. Multimodal physiological and flight-state data are first acquired and synchronized, then processed by an intelligent assessment layer to classify the pilot’s G-induced state. Based on the estimated risk level, the execution layer may initiate multi-zone anti-G suit inflation, pulsatile pressure compensation, or flight-control adjustment to reduce G load and stabilize flight conditions.

## 7 Research gaps and future priorities

Although the implementation technologies discussed above provide a pathway toward dynamic G-LOC risk prevention, several scientific and operational gaps remain before such systems can be reliably deployed in high-performance aviation. Future research should therefore move beyond demonstrating individual sensors, algorithms, or protective devices in isolation, and should focus on validating integrated mechanisms, improving generalizability, and ensuring safe human–automation interaction under realistic flight conditions.

### 7.1 Mechanistic uncertainty in rapid-onset G-LOC and recovery dynamics

Current understanding of G-LOC is still largely grounded in the classical framework of cerebral hypoperfusion. This framework explains the dominant pathway from +Gz exposure to reduced cerebral perfusion, neural impairment, and loss of consciousness. However, rapid-onset G-LOC, inter-individual variability, and delayed recovery of cognitive function indicate that this process cannot be fully explained by a single static perfusion threshold.

Future research should validate integrated models that couple cardiovascular dynamics, cerebral perfusion, cerebral oxygenation, neural functional integrity, and biomechanical effects under time-varying +Gz exposure (Morris et al., 2024). The role of intracranial mechanical stress under rapid-onset acceleration also requires further clarification. Although early studies and historical hypotheses suggested that intracranial pressure dynamics or inertial effects within cranial structures may influence cerebral circulation during gravitational stress (Henry et al., 1951), current evidence still indicates that cerebral hypoperfusion and insufficient cerebral oxygen delivery are the dominant explanatory mechanisms for G-LOC (Werchan, 1991; Whinnery and Forster, 2013). Intracranial mechanical effects should therefore be considered only as possible secondary contributors that may interact with hemodynamic delay and cerebrovascular regulation under specific acceleration profiles. Future studies should determine whether such effects can be measured under operationally relevant +Gz onset rates, whether they independently contribute to neural dysfunction, and how their contribution can be distinguished from reductions in cerebral perfusion pressure, cerebral blood flow, and cerebral oxygenation.

Potential physiological interventions also require stronger mechanistic validation. For example, brief exposure to mild hypercapnia, induced by breathing 5% CO_2_, has been reported to increase cerebral blood flow during lower-body negative pressure, suggesting that respiratory or gas-composition modulation may have potential to support cerebral perfusion under acceleration-related circulatory challenge (Harrell et al., 2024). However, its operational applicability, safety limits, and interaction with pressure breathing and AGSM remain uncertain. Therefore, future work should not only identify new intervention targets, but also determine when, for whom, and under what G-load profiles these interventions are effective.

### 7.2 Ecologically valid multimodal physiological datasets

A major limitation of current G-LOC prediction research is the restricted generalizability of centrifuge-derived findings to real operational aviation conditions. Although human centrifuge studies are indispensable, their generalizability to real flight conditions remains uncertain (Khan et al., 2025).

Several operational factors may alter physiological responses and G-LOC susceptibility. Real flight involves rapidly changing maneuver sequences, aircraft attitude changes, recovery intervals, and pilot control actions rather than isolated standardized +Gz profiles. At the same time, cognitive workload, decision-making demands, communication, threat monitoring, and aircraft-control tasks may affect autonomic activation, breathing strategy, AGSM execution, and behavioral performance. HR and HRV studies in fighter-pilot simulator tasks indicate that cardiovascular signals are sensitive to task demand and pilot mental workload (Mansikka et al., 2016). In addition, hypoxia, fatigue, dehydration, thermal stress, vibration, emotional stress, and mission pressure may reduce physiological reserve or increase response variability. Hypoxia combined with high cognitive load can alter autonomic responses in military personnel (Temme et al., 2023), fatigue is a major safety risk in aviation (Wingelaar-Jagt et al., 2021), and measurable fluid loss has been reported during military flight, particularly in fighter-jet operations (Levkovsky et al., 2018). Therefore, a physiological threshold or warning feature identified in centrifuge settings may not retain the same sensitivity, specificity, warning lead time, or false-alarm profile in operational aviation.

Operational translation is also constrained by measurement feasibility. In centrifuge studies, sensors can be placed under relatively controlled conditions and synchronized with known exposure profiles. In flight, wearable sensors must tolerate vibration, sweating, helmet and mask constraints, pressure breathing, aircraft motion, electromagnetic interference, and body movement during AGSM. These factors may introduce artifacts into ECG, PPG, EDA, EMG, respiratory, EEG, and fNIRS signals. Therefore, military aviation physiological monitoring requires sensors that are reliable in aerospace environments, compatible with flight equipment, and minimally intrusive to pilot performance (West, 2013; Shaw and Harrell, 2023). Recent wearable fNIRS work demonstrates the feasibility of monitoring cerebral oxygenation during high-G exposure, but further validation in operational environments remains necessary (Roumengous et al., 2024).

Future validation should follow a staged translational strategy. First, centrifuge studies should continue to provide mechanistic validation using synchronized +Gz profiles, cardiovascular variables, cerebral oxygenation, respiratory signals, EDA, EMG, and behavioral-performance measures. Second, centrifuge and flight-simulator paradigms should incorporate visual, cognitive, motor, workload, fatigue, and decision-making tasks to better approximate operational demands. Third, wearable physiological monitoring should be deployed during routine training flights or operationally representative sorties to collect ecologically valid data, including flight-state variables, maneuver profiles, pilot workload, environmental conditions, and synchronized physiological and behavioral signals. Recent in-flight stress-state modeling using pilot ECG, respiratory, and acceleration data illustrates the feasibility of collecting operational physiological datasets, although G-LOC-specific validation is still required (Shang et al., 2026).

Model validation should explicitly test transferability across centrifuge, simulator, and in-flight datasets. Algorithms developed in centrifuge environments should be externally evaluated using operationally meaningful metrics, including sensitivity, specificity, diagnostic accuracy, precision, recall, false-alarm burden, missed-detection risk, warning lead time, artifact robustness, missing-modality tolerance, uncertainty calibration, and cross-subject, cross-device, and cross-scenario generalizability. Because true G-LOC events are rare in operational flight, future studies should also define intermediate risk states, such as compensated high-G strain, visual impairment, A-LOC, degraded task performance, and delayed recovery. Centrifuge experiments and operational flight monitoring should therefore be treated as complementary sources of evidence: the former provides mechanistic control and safety, whereas the latter provides ecological validity and determines whether candidate indicators remain reliable and actionable under real mission constraints.

### 7.3 Validation of individualized monitoring and adaptive protection strategies

Although individualized monitoring may identify early physiological and functional degradation before complete G-LOC, its practical value depends on whether the resulting protective actions are timely, physiologically appropriate, and operationally safe. Previous work has suggested that physiological markers such as cerebral oxygenation may be useful not only for warning of impending incapacitation, but also for supporting adaptive protection when the pilot is unable to maintain effective control (Tripp, 2007). Warning-only approaches may be insufficient in some incapacitation scenarios, as illustrated by automatic recovery concepts such as Auto-GCAS (Suplisson, 2015). However, for a physiology-oriented validation framework, the primary focus should remain on whether monitoring-guided interventions improve physiological stability and functional recovery under high-G stress.

Future studies should therefore validate graded intervention strategies under realistic high-G task conditions. Key issues include the warning lead time required for effective pilot response, the acceptable balance between missed detections and false alarms, and the potential unintended effects of automated interventions such as G-load reduction, flight-envelope protection, pressure-breathing support, or temporary stabilization control.

Because these interventions may influence pilot behavior and aircraft operation, validation should also consider operational safety margins and unintended effects. Intervention logic should not introduce new hazards under uncertain flight conditions (Belcastro, 2012), and control-assistance strategies require evaluation in piloted simulation or flight-test settings to ensure that protective logic preserves controllability (Lombaerts et al., 2016). In the context of G-LOC prevention, these considerations should be treated as translational constraints rather than the central focus of the physiological model. Therefore, future validation should evaluate intervention timing, physiological effectiveness, task-performance outcomes, recovery quality, false-alarm tolerance, and robustness across different pilots and G-load profiles.

## 8 Conclusion

G-induced loss of consciousness has traditionally been interpreted as a physiological consequence of insufficient cerebral perfusion under +Gz acceleration. This review extends that view by conceptualizing G-LOC as a dynamic physiological state-transition process shaped by external G-load exposure, cardiovascular compensation, cerebral perfusion, neural function, and active countermeasure performance. From this perspective, G-LOC is not only a terminal loss-of-consciousness event, but also the endpoint of a progressive degradation process that may involve visual, cognitive, behavioral, and operational impairment before complete incapacitation occurs.

Future G-LOC prevention systems should therefore integrate physiological monitoring, predictive modeling, adaptive training, anti-G protection, pressure-breathing support, and human–machine intervention into a protection architecture. Such strategies should aim not only to prevent complete loss of consciousness, but also to preserve cerebral oxygenation, accelerate functional recovery, and maintain pilot operational performance during high-G exposure.
